# Case of Biliary Calculi, Complicated with Disease of the Heart

**Published:** 1853-06

**Authors:** Ira Manley

**Affiliations:** Markesan, Marquette County, Wisconsin


THE NORTH-WESTERN

MEDICAL AND SURGICAL JOURNAL.

NEW SERIES.

VOL II. JUNE, 1853. No. 2.

### ORIGINAL COMMUNICATIONS.

ARTICLE 1.—*Case of Biliary Calculi, complicated with disease of the Heart.* By IRA MANLEY, Jr., of Markesan, Marquette County, Wisconsin.

Oct. 30th, 1849.—Was summoned to visit Miss H. S., aged 24. Found her complaining of a violent pain under the edge of the ribs of the right side and also at the epigastrium, attended with vomiting.

From the history of the patient I learned that she had previously been similarly affected, the attacks occurring at intervals of about a month. She had formerly resided in Illinois where Ague and Fever were very common, she had suffered from Bilious Fever. Aside from the periodicity of the attacks, there was no symptom of uterine derangement. The pain in the side and vomiting did not occur at the menstrual period. The menses, with a single exception, occured quite regularly during the time of my acquaintance with the patient.

There was no excitement of the pulse, the papillæ on the tongue were raised and red, edges of the tongue red. Prescribed morphine, with sinapisms externally. Patient continued to vomit until a large quantity of bile was discharged.

Nov. 28.—Was called again and found a similar set of symptoms; taking the hint from the first occasion I gave Ipecacuan. to promote emesis, bile was rapidly discharged, and a full dose of morphine soon quieted the difficulty.

In the interval. prescribed Quinine, Strychnine, Fowler's solution, &c. The appetite was generally good and, aside from a dragging sensation and a tenderness in the right hypochondrium, she was tolerably comfortable.

She passed the interval for Dec. comfortably—1850. Jan. 8, found her suffering with an aggravation of all the old set of symptoms and in addition suppression of menses and a violent bronchitis; on the 16th she was convalescent, with return of the menses.

She had no other violent attack during the winter or spring. This exception we attributed principally to a frequent use of the comp. cathartic pill, with counter irritation. Still the side was troublesome. To my mind the precise pathological condition of the liver was not very clear, therefore I advised her to visit Chicago and get advice from Prof. Herrick. To him I wrote that I supposed from the tenderness, dragging, produced by motion, that the investing membrane or ligaments were the points implicated.

He prescribed Strychnine in solution with Sul. Acid, also Oleum Jec. Asselli, and externally, the Nitro Mur. Acid, Bath. She improved under his treatment. In May, 1851, she married, from which time till October she had but few returns of the vomiting.

Oct. 11.—Found her coughing and expectorating frothy mucus tinged with blood. Knowing that except occasional bronchital irritation her lungs were quite healthy, I at once suspected the heart to be in trouble. Physical signs soon proved to me that the right side of the heart had enlarged by thining of its walls.—A recurrence of the vomiting together with palpitation of the heart soon became very distressing. While waiting for Cyanuret of Potass. for which we immediately sent, we used the cold bathing; this gave partial relief, but after a few trials ceased to give any whatever. The Pruss. Acid was more useful, as for almost a year it was the only agent except chloroform which was reliable to quiet the action of the heart, and at the same time allay the irritability of the stomach. During the repeated attacks she suffered the following winter, nothing answered so well to quiet the anguish, which was intense.

During the summer of 1852 she again visited Chicago. In addition to my diagnosis, Prof. Herrick diagnosed granulations of the edges of the valves. He gave her no hope. It was he who gave her chloroform. This was principally to allay the suffering occasioned by distention of the different cavities. Edœma showed itself first in the lower extremity, from which general anasarca ensued, and when the Prof. saw her, the thorax and abdomen were filling up with effused fluid. I should not have ventured the chloroform on account of the heart disease, but when she returned I gave it to her in large quantities, after all medicine given by the mouth was rejected; the effect for the time was very grateful to the patient. He also prescribed Citrate of Pota-s. with an excess of Citric acid. This for a while produced rapid absorbtion of the effused fluids, but like all other remedies it ceased to benefit. She filled up again, and died in November.

Autopsy. J. Chapel, Esq., present.

Raised the Sternum, Lungs appear about one half collapsed, fluid occupying their place, Lungs somewhat congested, some adhesions of the left Lung, no tubercle. Pericardium contains about 6 oz. serum. Cava greatly dilated, right auricle about the size of a man's hand, closed walls extremely thin, musculi pectinati appeared very strong. Foramina hebesii normal.

The auriculo-venticular opening allows the passage of two fingers. The edges of the tricuspid were granulated and could not have been approximated, The granules were small. Right Ventricle greatly enlarged, depth five inches. Walls 4 lines three-eights inches thick. Carneæ columnæ much hypertrophied, Semilunar Valves of pulmonary artery healthy. Left Auricle nearly normal, Auriculo-Venticular orifice will not admit the end of the little finger. Mitral Valves granulated, similar to the tricuspid. One of the Semilunar valves of Aorta slightly granulated No trace of ossification. Heart weighed 25 oz.

Liver weighed 4 lbs. 2 oz. Left lobe firmly attached to the spleen. From its mottled appearance: judged this lobe had suffered congestion.

The walls of the gall-bladder were much thickened, the fundus and neck each contained a *concretion*, something larger than a rifle ball. The body of the bladder formed an hour glass contraction.

The duct barely allowed the passage of a probe, showing that no concretion had passed; there was in the cyst, but a small quantity of mucus.

The uterus and its appendages appeared normal. The cavity of the abdomen contained a large quantity of fluid.

It was observed throughout her long course of bad health that acids were better borne and more servicable to quiet the stomach than any other form of medication.

Had the contracted condition of the left auriculo-ventricular orifice anything to do with the production of this diseased heart? If so, should the left auricle have suffered as well as the right?

Prof. H. says there was no disease of the heart in 1850, and I am of the same opinion; but there was effusion at that time. Did this effusion originate in the liver? what effect had it in the production of the heart disease? or had the frequent vomiting for so great length of time any agency in the matter?

